# One-Step Synthesis of Aminobenzoic Acid Functionalized Graphene Oxide by Electrochemical Exfoliation of Graphite for Oxygen Reduction to Hydrogen Peroxide and Supercapacitors

**DOI:** 10.3390/molecules27217629

**Published:** 2022-11-07

**Authors:** Yuting Lei, Ludmila dos Santos Madalena, Benjamin D. Ossonon, Fausto Eduardo Bimbi Junior, Jiyun Chen, Marcos R. V. Lanza, Ana C. Tavares

**Affiliations:** 1 Institut National de la Recherche Scientifique—Énergie Matériaux Télécommunications (INRS-EMT), 1650 Boulevard Lionel-Boulet, Varennes, QC J3X 1P7, Canada; 2 Instituto de Química de São Carlos, Universidade de São Paulo, Av. Trab. São Carlense, 400—Parque Arnold Schimidt, São Carlos 13566-590, SP, Brazil

**Keywords:** electrochemical exfoliation of graphite, amine oxidation, graphene oxide, aminobenzoic acid, oxygen reduction reaction, supercapacitors

## Abstract

Graphene-based materials have attracted considerable attention as promising electrocatalysts for the oxygen reduction reaction (ORR) and as electrode materials for supercapacitors. In this work, electrochemical exfoliation of graphite in the presence of 4-aminebenzoic acid (4-ABA) is used as a one-step method to prepare graphene oxide materials (EGO) functionalized with aminobenzoic acid (EGO-ABA). The EGO and EGO-ABAs materials were characterized by FT-IR spectroscopy, X-ray photoelectron spectroscopy, Raman spectroscopy, X-ray diffraction and scanning electron microscopy. It was found that the EGO-ABA materials have smaller flake size and higher density of oxygenated functional groups compared to bare EGO. The electrochemical studies showed that the EGO-ABA catalysts have higher activity for the ORR to H_2_O_2_ in alkaline medium compared to EGO due to their higher density of oxygenated functional groups. However, bare EGO has a higher selectivity for the 2-electron process (81%) compared to the EGO-ABA (between 64 and 72%) which was related to a lower content of carbonyl groups. The specific capacitance of the EGO-ABA materials was higher than that of EGO, with an increase by a factor of 3 for the materials prepared from exfoliation in 5 mM 4-ABA/0.1 M H_2_SO_4_. This electrode material also showed a remarkable cycling capability with a loss of only 19.4% after 5000 cycles at 50 mVs^−1^.

## 1. Introduction

Graphene-based materials have been extensively investigated because of their tunable chemistry (e.g., surface functional groups and heteroatoms doping), high surface area and good electrical conductivity [1,2]. Graphene oxide (GO) materials are abundant in surface functional groups such as hydroxyl, epoxy, carbonyl and carboxyl, and can be easily functionalized with other molecules or nanoparticles to form new compounds and composites with improved properties [3,4,5,6]. For these reasons GO and its composites have been used in a broad range of applications [7,8,9], including (bio)sensors [4,5], electrogeneration of hydrogen peroxide [10,11] and supercapacitors [1]. 

Hydrogen peroxide (H_2_O_2_) is used in wastewater treatment, disinfection, and pulp- and paper bleaching [10,11,12]. The electrochemical synthesis of H_2_O_2_ via the oxygen reduction reaction (ORR) is considered a cleaner and more sustainable process compared to the conventional antraquinone one [10,13]. In alkaline medium, the reduction of O_2_ to H_2_O_2_ occurs according to Equation (1). Carbon materials, such as certain types of carbon blacks [14], oxidized carbon black [15,16], and oxidized carbon nanotubes [17], reduced graphene oxide (rGO) [18,19] are active and selective for ORR to H_2_O_2_ in alkaline electrolytes, and their activity and selectivity have been correlated to the amount of oxygen functional groups [16,17,19,20]. In addition, the carbon atoms adjacent to COOH [16,17,19] and C-O-C [17,19,20] were identified as the active sites with high selectivity for the 2-electron process, whereas carbons sites next to C=O were found bind too strongly to the peroxide and to promote its reduction to OH^−^ [16] (Equation (2)).

(1) $$O_{2}+H_{2}O+2{\;e}^{-}\;\to {\mathrm{HO}}_{2}^{-}+{\mathrm{OH}}^{-}\;E^{0}=-0.065\;V\;\left(\mathrm{vs}.\;\mathrm{SHE}\right)$$

(2) $${\mathrm{HO}}_{2}^{-}+H_{2}O+2{\;e}^{-}\;\to 3{\;\mathrm{OH}}^{-}\;\;E^{0}=+0.867\;V\;\left(\mathrm{vs}.\;\mathrm{SHE}\right)$$

Supercapacitors (SC) are charge storage systems characterized by high-power density, rapid charge–discharge, are environmentally friendly because of low CO_2_ emissions and have longer cycle life than batteries [1,21,22]. They can be used as complementary devices to conventional batteries in applications that require peak power pulses [21,23], or to power stretchable and wearable electronic devices [24]. Pseudocapacitors, a specific type of SCs, are based on charge storage involving fast surface redox reactions at the electrode/electrolyte interface [21,24,25], and GO materials are of potential interest as electrode materials for this technology [26]. The presence of functional oxygenated groups on the GO’s surface impedes the restacking of the layers and the agglomeration of the flakes, an important drawback associated with the use of graphene sheets [27]. These functional groups also improve the wettability of the electrode by the electrolyte and can participate in reversible redox processes thus increasing the pseudocapacitance [1,28]. However, the oxygenated functional group lowers the electrical conductivity of the GO materials so their content should be optimized [1].

Generally, GO materials are prepared using chemical methods such as the modified Hummers method [29], followed by a thermal treatment [19,30], by electrochemical reduction [31] or by hydrothermal treatment [18] to partially remove the functional groups and to obtain reduced graphene oxide (rGO). Electrochemical exfoliation of graphite is an environmentally friendly and low-cost method that offers the possibility of synthesizing GO materials in few hours [32,33,34,35]. Materials, usually referred as electrochemical exfoliated graphene oxide (EGO), with different properties (amounts and types of functional groups, density of defects, number of layers, flake sizes) can be easily synthesized by varying the experimental conditions during the electrochemical exfoliation, such as applied voltage and electrolyte [35]. Compared to the chemical methods (e.g., Hummers’ method: C/O ≈ 2) [29], the EGO materials obtained this way have a lower density of oxygenated functional groups (C/O > 4) [35] which could be of advantage for electrochemical applications [19,36]. In addition, the method also allows the preparation of EGO-based composites [37,38] or the surface modification of EGO with other molecules in a one-step process [39,40]. 

**Scheme 1:**
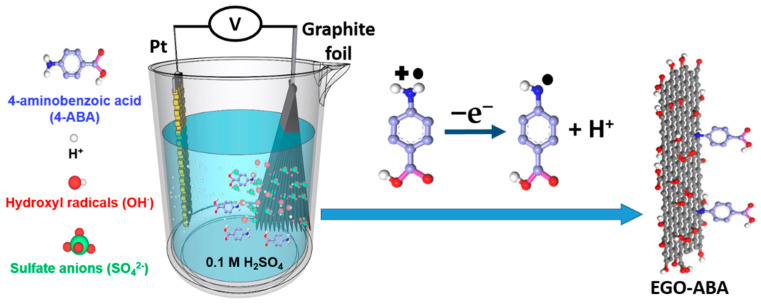
One-step synthesis of electrochemically exfoliated and aminobenzoic acid functionalized graphene oxide (EGO-ABA). The exfoliation of graphite is carried out in 0.1 M H_2_SO_4_ with the presence of 4-aminobenzoic acid.

In this work, electrochemical exfoliation of graphite in 0.1 M sulfuric acid containing 4-aminobenzoic acid (4-ABA) was conducted aiming at obtaining a series of aminobenzoic acid functionalized EGO materials (EGO-ABA). Modification (electrografting) of carbon surfaces by amine oxidation is a known method [6,41], and 4-ABA functionalized GO materials prepared by 2 steps methods were used in sensing and membrane applications [42,43,44]. However, little is known of its use during the electrochemical exfoliation of graphite to obtain functional graphene oxides. Considering the simplicity of the method and the large possible combinations of electrolytes and molecular precursors, graphene-type materials with diversified structures and compositions can be obtained and their properties should be investigated. During this one-step process, graphite is electrochemically oxidized and exfoliated to form EGO sheets, while the amine groups of 4-ABA are oxidized to radicals, followed by the formation of covalent bonds between the sp^2^ carbons (on the EGO basal plan) and the amine-derived radicals (Scheme 1) [6,41,45]. As will be shown, the amount of 4-ABA in the electrolyte alters significantly the exfoliation process, the composition and flake size of the EGO-ABA materials. Their electrochemical behavior and the role of the functional groups towards the ORR and the supercapacitors are investigated. The materials obtained from exfoliation in 4-ABA/H_2_SO_4_ solutions with [4-ABA] = 5 and 10 mM have the highest specific capacitance and the best activity for ORR, respectively. However, the selectivity of EGO for the 2-electron process decreases after functionalization with the aminobenzoic acid.

## 2. Results

### 2.1. Electrochemical Exfoliation of Graphite in the Presence of 4-Aminobenzoic Acid

In this work the electrochemical exfoliation of graphite was conducted with a two-electrode system where the graphite foil was used as the anode. The electrochemical cell was filled with 0.1 M H_2_SO_4_ electrolyte, and the concentration of 4-ABA varied between 0 and 40 mM. When a potential difference is applied between the electrodes, water is reduced at the cathode generating H_2_ and hydroxyl radicals are formed at the anode. These radicals attack the graphite electrode, facilitate the intercalation of ions from electrolyte within the graphitic layers, and the gases released (SO_2_, CO_2_) during the exfoliation assist the formation of EGO and EGO-ABA flakes [32,33,45].

**Scheme 2:**
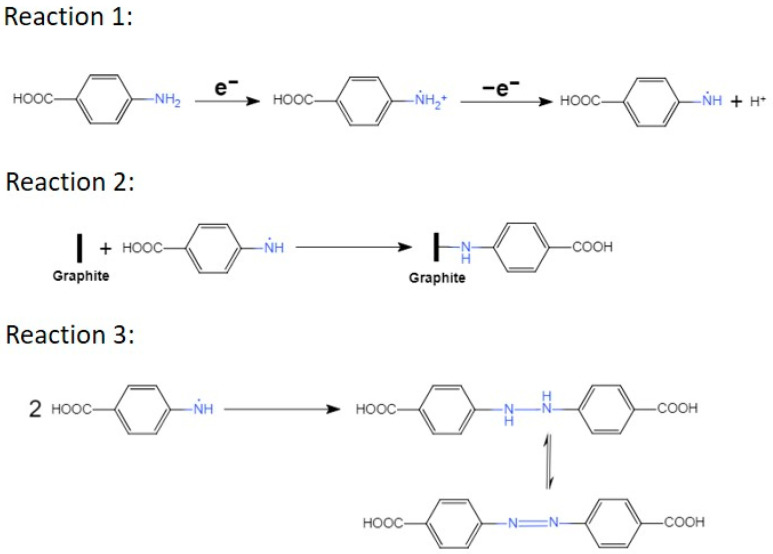
The chemical reactions describing the grafting of aminobenzoic acid on the basal plane of the graphitic layers and the formation of an azo compound.

However, the presence of 4-aminobenzoic acid (4-ABA) has a profound impact on the exfoliation process (rate and duration), including changes in color of the electrolyte. As the concentration of 4-ABA in the electrolyte increases, the lower the value of the initial current, the faster the electrolyte turns purple around graphite electrode until it finally became brown, and the longer the exfoliation time (Table 1). The reduction in the initial current value results from a higher electrolyte resistance due to the addition of 4-ABA. This translates in a lower concentration of hydroxyl radicals and in a slower intercalation of the sulfate anion between the graphite layers. Moreover, as shown in Scheme 2, the oxidation of 4-ABA (Reaction 1) competes with the oxidation of graphite and may also slowdown the exfoliation process. While the electrolyte coloration proves that the 4-ABA is being oxidized, it also indicates that the formation of an azo compound is taking place during the exfoliation and is competing with the functionalization of 4-ABA on the basal plan of EGO (Reaction 2 and 3) [41,46].

**Figure 1:**
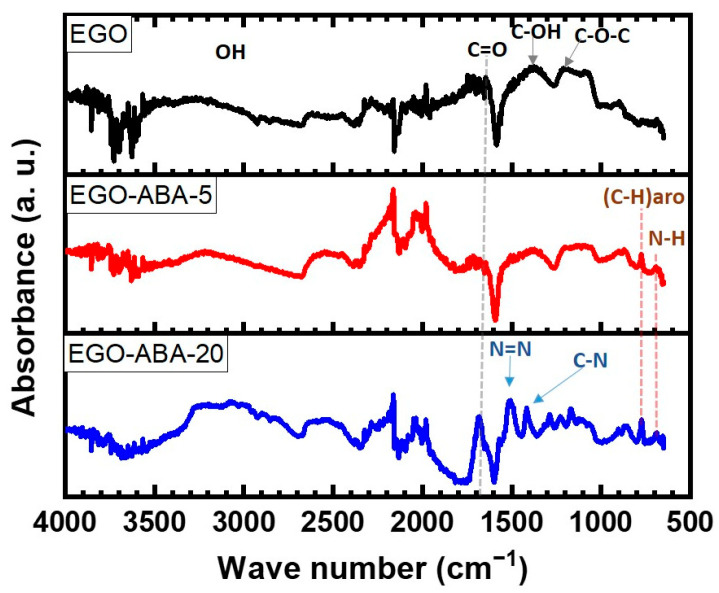
FT-IR spectra of EGO, EGO-ABA 5 and 20 materials.

Figure 1 shows the Fourier Transform Infrared (FT-IR) spectra of the EGO, EGO-ABA-5 and EGO-ABA-20. The spectrum of EGO displays the bands associated with C-O-C (1284 cm^−1^), C-OH (1370 cm^−1^), C=O (1680 cm^−1^) stretching vibration and a broadband coming from OH stretching vibration around 3000 cm^−1^ [39,43]. The peaks corresponding to C-H aromatic bending modes (770 cm^−1^) and N-H deformation vibration (secondary amine, at 700 cm^−1^) appear in the spectra of EGO-ABA samples and their intensity increases with the 4-ABA concentration [47,48]. The same trend is found for the C=O band. Additionally, for EGO-ABA-20 and 40 (Supplementary Figure S1a), the peaks corresponding to C-N stretching vibration (around 1410 cm^−1^) and to azo compounds N=N stretching vibration (1516 cm^−1^) are observed [49]. Finally, the peaks corresponding to asymmetric and symmetric NH_2_ stretching vibrations (primary amine, around 3400 cm^−1^, see FTIR spectrum of 4-ABA in Supplementary Figure S1b) are absent from the spectra of the EGO-ABAs materials [43,47]. FT-IR analysis confirms that the EGO was functionalized with 4-ABA, and that azo compounds were formed when the concentration of 4-ABA was above 20 mM.

**Figure 2:**
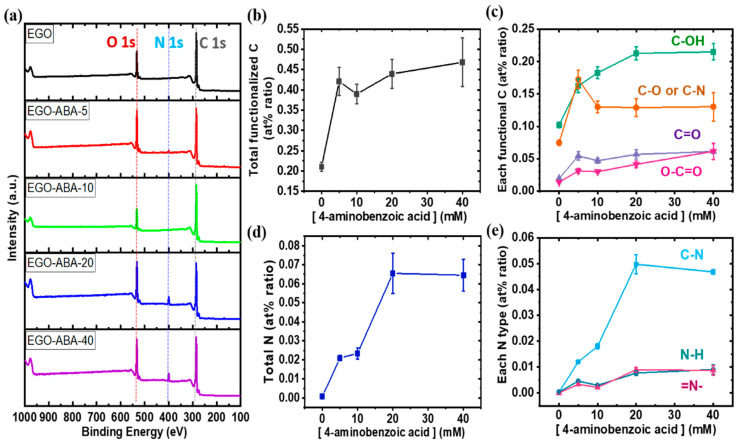
(**a**) XPS survey spectra; atomic percentage (at% ratio) of (**b**) total functionalized carbon, (**c**) each functional group, (**d**) total nitrogen and (**e**) each nitrogen type.

To get insights on the functionalization of EGO by 4-ABA, XPS was used to analyze the surface composition of the materials. The XPS survey spectra (Figure 2a) show the expected C 1s, N 1s and O 1s peaks at 285, 400 and 532 eV, respectively; being the N 1s peak more intense for EGO-ABA-20 and EGO-ABA-40. As shown in Supplementary Figure S2a, the C 1s spectrum was deconvoluted into five peaks identified as graphitic carbon: C-C and C=C (284.5 eV), C-OH (285.8 eV), C-O or C-N (286.8 eV), C=O (288 eV), and O-C=O (289 eV) [50,51]. Generally, the hydroxyl (C-OH) are on the basal plan and at the edges, while, the epoxy groups (C-O-C) are on the basal plan, the carbonyl (C=O) and carboxyl (O-C=O) groups are at the edges sites [52]. However, it should be noted that the grafting of 4-ABA which contains carboxyl (O-C=O) groups occurs on the basal plan of EGO [39,41]. The N 1s spectrum (Supplementary Figure S2b) was fitted into 3 peaks: C–N (399.8 eV), N–H (401.7 eV) and =N- (398.6 eV) [51,53]. The presence of =N- groups confirms that the 4-ABA underwent a side reaction leading to the formation of diazocompounds. The various functional groups and related carbon and nitrogen species were quantified, and the atomic percentages (at%) are presented in Figure 2b–e. As expected, these at% ratios increase with the increasing concentration of 4-ABA in the electrolyte, pointing for a successful functionalization of the EGO surface with 4-ABA, but reached a plateau for [4-ABA] ≥ 20 mM. However, all plots—except the one for C-OH—have an inflexion point at 10 mM of 4-ABA. Looking at Figure 2d,e, it can be concluded that most of the N species are in the form of N-C bonds with small amounts from N-H and =N- bonds. In addition, the at% ratios of other C and N-species decrease from 5 to 10 mM 4-ABA, being the C-OH/C-N species the only exception. These trends suggest that the functionalization of EGO by oxidative grafting of 4-ABA dominates up to 10 mM, after which the functionalization of EGO through physical adsorption of 4-ABA and the diazo compounds dominates. Hence, 5 to 10 mM of 4-ABA seem to be the appropriate concentration to conduct simultaneously the graphite exfoliation and 4-ABA grafting on the EGO surface, while too much 4-ABA increases the occurrence of side reactions and slows the exfoliation process.

**Figure 3:**
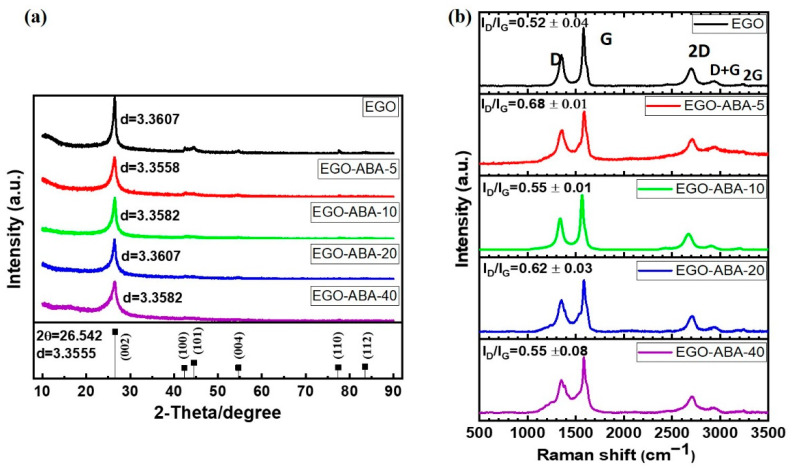
(**a**) The powder XRD patterns of the EGO and EGO-ABA materials. The position of the diffraction peaks of graphene (PDF 65-6212) is included in the lower panels for reference, respectively. (**b**) Raman spectra of the EGO and EGO-ABA materials.

The bulk structure of EGO and EGO-ABA materials was characterized by XRD. As expected, the XRD patterns (Figure 3a) show the typical features characteristic of graphene materials with a high intensity diffraction peak at 26.5° attributed to the (002) plane of graphite [35,45]. The position of the diffraction peak does not vary significantly with the extent of functionalization, and it corresponds to an average lattice spacing of 0.3587 nm. The lattice spacing value and the absence of the graphene oxide’s diffraction peak (usually at 10°) [54] suggest that the EGO materials have a low degree of oxidation and that the 4-ABA is on the external surface of the EGO flakes [45].

Raman spectroscopy was used to characterize the structural defects of the EGO and EGO-ABA materials in the form of free-standing films. For each sample, five Raman spectra were recorded, their average I_D_/I_G_ ratios and representative spectra show in Figure 3b. The strong G band around 1600 cm^−1^ corresponds to the sp^2^ carbon network [55]. The D band at about 1350 cm^−1^ is related to defects such as edge defects, vacancies, etc [56]. The 2D band which is related to the number of layers of graphene materials shifts from 2687 cm^−1^ to 2711 cm^−1^ (Supplementary Table S2) suggesting these samples have few layers (≤6 layers) [57]. Low-intensity D + G (≈2950 cm^−1^) and 2G (≈3248 cm^−1^) bands were found proving that these graphene sheets are slightly oxidized [58]. Finally, the ratio between the intensity of the D and G bands (I_D_/I_G_) was calculated, being 0.52 ± 0.02 for EGO and varying between 0.68 ± 0.01 for EGO-ABA-5 and 0.55 ± 0.08 for EGO-ABA-40. Based on these results, it can be concluded that EGO-ABA materials have more defects than EGO, and that EGO-ABA-5 seems to have the highest number of defects. These results are consistent with the other experimental findings indicating that the oxidative grafting of 4-ABA is favored at low concentration of the amine and that the presence of 4-ABA in the electrolyte hinders the oxidation and exfoliation of graphite (Table 1).

All samples exhibited the typical morphology of graphene materials [39], characterized by curled and overlapped flakes, Supplementary Figure S3. However, the sheet size of EGO is larger than that of EGO-ABA. This may be explained by the slow kinetics of exfoliation in the presence of 4-ABA, where the slow intercalation of the sulfate anions between the graphite sheets combined with the evolution of SO_2_, CO_2_ gases during the exfoliation [32] lead to the formation of the EGO-ABA with smaller flakes [35].

### 2.2. Electrochemical Characterization of EGO and EGO-ABA Materials and Their Performance for the and Oxygen Reduction to Hydrogen Peroxide in 0.1 M KOH

**Figure 4:**
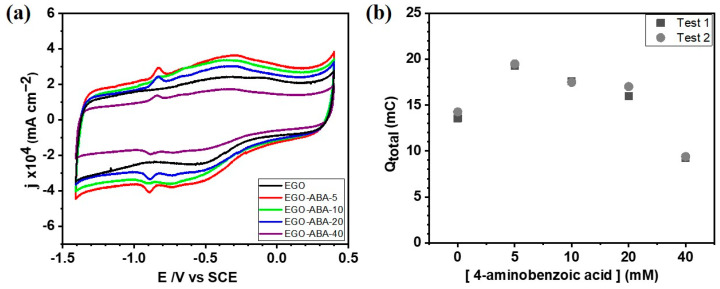
(**a**) Cyclic voltammetry of the EGO and EGO-ABAs modified electrodes in N_2_-saturated 0.1 M KOH solution after 20 cycles, scan rate 50 mV s^−1^. (**b**) The total charge (Q_total_) calculated from the voltammograms presented in Supplementary Figure S4a.

Cyclic voltammetry in 0.1 M KOH was employed to evaluate the electrochemical behavior of EGO and EGO-ABA thin layers deposited on the surface of a glassy carbon electrode (GCE). As shown in Figure 4a and in Supplementary Figure S4a, the CV of EGO/GCE in N_2_-saturated 0.1 M KOH has only one broad cathodic peak (~−0.30 V) and one broad anodic peak (~−0.50 V) corresponding to reversible redox reactions of oxygen functional groups; while the CVs of EGO-ABAs/GCE have an additional narrow and well defined pair of anodic and cathodic peaks (~−0.90 V and −0.85 V, respectively) corresponding to oxidation/reduction of secondary amine groups (-NH-) [59]. This was confirmed by recording CVs of the GCE in N_2_-saturated 0.1 M KOH with different concentrations of 4-ABA. As shown in Supplementary Figure S4b, no redox peaks were found indicating that 4-ABA is not electroactive in these conditions. The total voltammetric charge (Q_total_) was plotted as a function of [4-ABA] as shown in Figure 4b. It can be appreciated that the EGO-ABA-5 based electrode has the highest Q_total_ indicating that this material has the largest electrochemical surface area. A similar trend was found for the Faradic charge (Q_Faradic_) as shown in Supplementary Figure S5.

**Figure 5:**
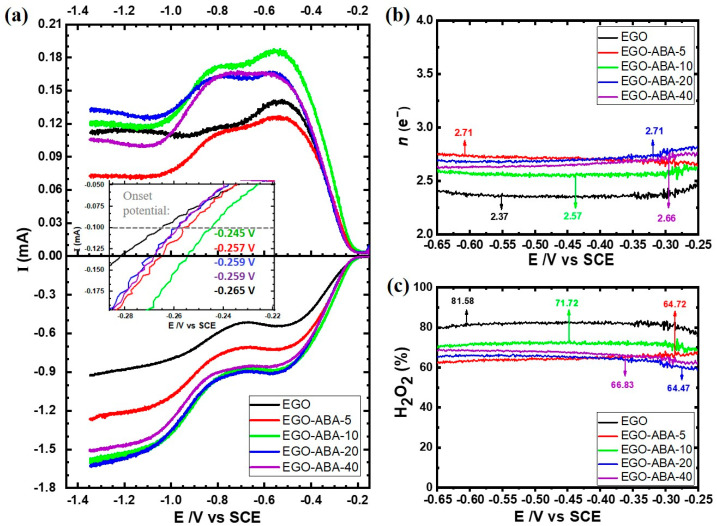
(**a**) Linear sweep voltammograms for the oxygen reduction reaction for EGO and EGO-ABA electrodes 0.1 M KOH at 5 mV s^−1^ with a rotation of 1600 rpm: disc current (bottom panel) and ring currents (top panel). The potential dependence of (**b**) number of transferred electrons (*n*) and (**c**) of the yield of hydroperoxide formation on EGO and EGO-COOHs based electrodes.

To evaluate in detail, the electrocatalytic activity and selectivity of EGO and EGO-ABAs for ORR, a set of the rotating ring-disc electrode (RRDE) polarization curves were recorded in O_2_ and N_2_ saturated 0.1 M KOH. The background subtracted disk and ring currents are shown in the bottom and top panels, respectively, of Figure 5a. The disk currents consist of two reduction waves, the first one having a limiting current plateau between −0.50 ~−0.85 V and the second one starting at more negative potentials (around −1.00 V). These LSVs are characteristic of carbon materials [60] and indicate that ORR is proceeding through the 2-electron process with the formation of hydroperoxide anion (Equation (1)) followed by a second 2-electron process leading to the formation of the hydroxyl anion (Equation (2)).

The onset potential, here defined as the potential necessary to reach current of −0.1 mA, is more positive for EGO-ABA compared to EGO indicating a higher ORR activity after functionalization, Figure 5a (insert). More specifically, the EGO-ABA-10 has the most positive onset potential. Moreover, the current intensity also increases from EGO to EGO-ABA-10, decreasing afterwards, confirming the highest ORR activity for this catalyst. The trend found for the activity seems to follow the one found for Q_Faradic_, which agrees with previous reports in the literature that have correlated the ORR activity to H_2_O_2_ of carbon materials with the content of oxygenated functional groups [16,17,19,20]. The higher activity of EGO-ABA is clearly related to a higher amount of COOH, C=O and C-O-C functional groups (and density if the smaller size of the flakes is taken into consideration) compared to EGO (see Figure 2).

The selectivity of the EGO and EGO-ABA catalysts toward the electrogeneration of H_2_O_2_ was evaluated through the analysis of the disk and ring currents, and Equations (3) and (4) were used to estimate the number of exchanged electrons (*n*) during ORR and the peroxide species (HO_2_^−^) yield, respectively:

(3) $$n_{e^{-}}=4\cdot \frac{i_{d}}{i_{d}+\frac{i_{a}}{N}}$$

(4) $$H_{2}O_{2}\;\left(\%\right)=\;\frac{2i_{a}/N}{i_{d}+i_{a}/N}\cdot 100\%$$

where *i_d_* and *i_a_*, are the disc and ring current, respectively, and *N* is the collection efficiency (*N* = 0.37).

The *n* value is close to 2.4 for EGO and it increases up to 2.7 for EGO-ABA, Figure 5b. This indicates that the ORR proceeds predominately via 2e^−^ pathway with the production of HO_2_^−^ on both EGO and EGO-ABA. However, and as shown in Figure 5c, the selectivity for H_2_O_2_ decreases significantly after the surface modification of the EGO with 4-ABA, according to the following trend: EGO (81.6%) > EGO-ABA-10 (71.2%) > EGO-ABA-5 ≈ EGO-ABA-20 ≈ EGO-ABA-40 (64–66%). The high selectivity of EGO is most probably associated with the relatively low amount of C=O groups [17,61] on large size flakes also characterized by a low density of defects on the basal plane. As the amount and density of C=O groups increases in the EGO-ABA series, the selectivity decreases.

The EGO prepared in this work has a much higher selectivity for H_2_O_2_ than Printex L6 (63.4% of H_2_O_2_ yield) [18], rGO obtained by Hummers method followed by solvothermal reduction at 150 °C (72.9%) [18], and graphene obtained by Hummers method followed by high temperature reduction at 1050 °C in Ar (55%) [60]. It has however a lower selectivity than mild reduced graphene oxide (mrGO, ≈100%) prepared by the Hummers’ method and reduced at 100 °C under N_2_ flow [19] and oxidized carbon nanotubes (O-CNTs, 90%) [17].

### 2.3. Performance of EGO and EGO-ABA Materials for Supercapacitors

**Figure 6:**
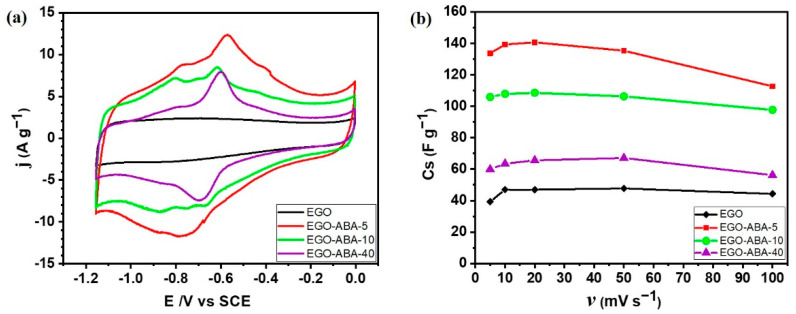
(**a**) Cyclic voltammograms (CV) of graphene materials, before (EGO) and after surface functionalization (EGO-ABA) collected at the scan rate of 50 mV s^−1^ from 0 to −1.15 V. (**b**) Specific capacitance (C_s_) versus the scan rate (υ) for EGO and EGO-ABA based electrodes.

Figure 6a shows the cyclic voltammograms of EGO and EGO-ABA electrodes in 6 M KOH collected at the scan rate of 50 mV s^−1^ from 0 to −1.15 V. The cyclic voltammogram of the Ni foam support is shown in Supplementary Figure S6. The EGO-ABA materials show a large current response with pronounced redox peaks in the potential range from −0.3 to −1 V related to the oxidation and reduction of functional groups, as previously mentioned in Section 3.2. These related redox reactions can be expressed as follows [62]:

(Reaction 4) $$-\mathrm{COOH}+{\mathrm{OH}}^{-}↔-\mathrm{COO}+H_{2}O+e^{-}$$

(Reaction 5) $$>C-\mathrm{OH}+{\mathrm{OH}}^{-}↔>C=O+H_{2}O+e^{-}$$

(Reaction 6) $$>C=O\;+{\;e}^{-}\;↔{\;>C-O}^{-}$$

The current intensity is the highest for EGO-ABA-5 and then it decreases for EGO-ABA-10 and EGO-ABA-40. The peaks are less visible on the unmodified EGO which shows a quasi-rectangular-shaped and symmetric cyclic voltammogram typical of a double layer capacitive behavior. First, the low content of 4-ABA in the reaction medium could lead to a uniform grafting of ABA molecules on EGO and could suppress the aggregation of the graphene oxide nanosheet and facilitate the redox reactivity, as illustrated in Figure 6a for EGO-ABA-5. Additionally, it could enhance the surface wettability and the accessible surface area of the electrode leading to a higher pseudocapacitance, as illustrated in Figure 5a. However, the excessive amount of 4-ABA during the synthesis lead to reaction between amines and the adsorption of these products on the EGO surface. This hinders the porosity path within the graphene sheets, is unfavorable to the rapid transport of electrolyte ions and even negatively affects the overall electrochemical performance of the samples, as shown in Figure 6a for EGO-ABA-40. 

Cyclic voltammograms were also recorded at different scan rates (Supplementary Figure S7) to determine the specific capacitance of each electrode. Figure 6b displays the variation of specific capacitances which decrease gradually with increasing scan rate [38]. However, the capacitances of EGO and EGO-ABA-5 at 5 mV s^−1^ are lower than those at 10 and 20 mV s^−1^, which is unusual, indicating that the electrodes’ surface area is less accessible at the lowest scan rate. Other studies have reported similar trends with carbon materials and it was proposed that the structure of the materials collapses when scanning at low scan rate or charging at low current density [63,64]. Furthermore, the specific capacitances of EGO-ABA-5 and EGO-ABA-10 are much larger than that of EGO-ABA-40 and of EGO. As already observed with the voltammetric charges obtained with 0.1M KOH (Figure 4b), the capacitance of the EGO-ABA materials is higher than that of EGO and then gradually decreases with increasing of the amine concentration in the reaction medium from 5 to 40 mM. The oxygen functional groups (Figure 3c) besides participating in the fast redox processes that provide pseudocapacitance [54], play a very important role in the charging/discharging processes because they render the internal surface of graphene matrix accessible to the ions in the electrolyte [54,55].

**Figure 7:**
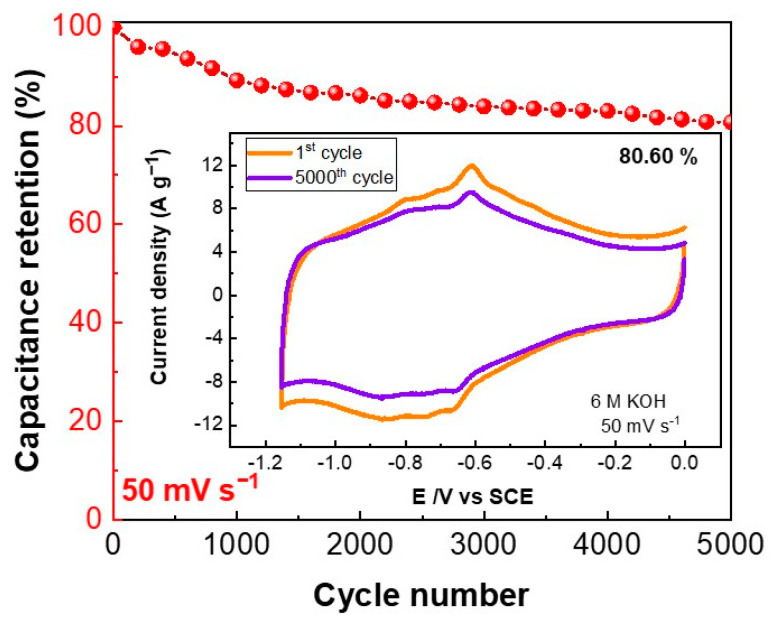
Cycling performance of the EGO-ABA-5/NF electrode at 50 mV s^−1^ for 5000 cycles; the inset shows CV curves for the 1st and 5000th cycles.

Extended cycling capability is a key parameter of supercapacitors. Hence, the performance of the EGO-ABA-5 electrode was evaluated by recording up to 5000 cycles at 50 mV s^−1^. As shown in Figure 7, the electrode retained 80.6% of the initial capacitance after 5000 cycles in 6 M KOH. This decrease of capacitance can be attributed to desorption of the non-covalently attached ABA molecules that are loosely bound to the EGO surface. [56,57]. In any case, the extended cycling test demonstrates the remarkable electrochemical durability of this electrode material and its potential to be used in supercapacitors.

## 3. Materials and Methods

Details related to materials, instrumentation, and physicochemical characterization techniques can be found in the Supplementary Materials (SM).

### 3.1. Preparation of Aminobenzoic Acid Functionalized Graphene Oxide (EGO-ABA)

In a two-electrodes system, a graphite foil (5 cm × 2 cm × 0.05 cm) was used as anode and a Pt mesh (6 cm^2^) was used as cathode. Both electrodes were immersed into 0.1 M H_2_SO_4_ electrolyte with different concentrations of 4-aminobenzoic acid (4-ABA, 0, 5, 10, 20, 40 mM) and connected to the direct current power supply. The distance between the two electrodes was 6 cm. After applying a potential difference of 8 V, the electrochemical exfoliation of graphite started immediately. After the exfoliation, the EGO sheets were washed with Millipore water and collected by vacuum filtration through an MF-Millipore membrane filter with 0.22 μm pore size. pH paper was used to verify the presence of residual acid. After several washes with water and vacuum filtration, the pH of the colorless filtrate was found to be around 5–6, indicating that the sulfuric acid electrolyte and the adduct produced by the side reactions have been removed. 

The EGO and EGO-ABA powders were then dispersed in water by ultrasonication for 90 min to maximize the exfoliation. The dispersion was freeze-dried, and the powders stored for further use. The samples are named according to the concentration of 4-aminobenzoic acid, such as EGO (0 mM of 4-ABA) and EGO-ABA-5 (5 mM of 4-ABA). Table 1 summarizes the experimental observations during the synthesis of the EGO materials, including the initial current, the exfoliation time, and the color of electrolyte. 

### 3.2. Preparation and Electrochemical Characterization of EGO-ABA Based Electrodes for ORR Study

Electrochemical studies in 0.1 M KOH were conducted on glassy carbon electrodes (GCE) disk and on a glassy carbon rotating disk ring electrode (RRDE) covered with EGO or EGO-ABA catalyst layers. Catalyst inks were prepared by dispersing 10 mg of EGO or EGO-ABA flakes in 525 μL isopropyl alcohol and 25 μL of 5 wt% Nafion^®^ perfluorinated resin solution. Subsequently, the inks were sonicated for 1 h. The electrodes (diameter of 5 mm) were polished on suede with 0.3 and 0.05 μm Al_2_O_3_ powder, rinsed with Millipore water and washed thoroughly in an ultrasound bath. Next, the disks were covered with an appropriate volume of catalyst ink to obtain a catalyst loading of 463.8 μgcm^−2^, and dried in room temperature. The GCE/RRDE electrodes covered with EGO materials, a counter electrode (Pt coil), and a saturated calomel reference electrode were all housed in a single compartment electrochemical cell.

Cyclic voltammetry was first conducted on the GCE in saturated N_2_ electrolyte at 50 mV s^−1^ from 0.4 V to −1.4 V vs. SCE and 20 cycles were recorded for each material. For the ORR studies, the RRDE and the following protocol were used: (i) three cyclic voltammograms from 0.4 V to −1.4 V vs. SCE were recorded at 50 mVs^−1^; (ii) linear sweep voltammograms (LSV) between −0.10 V to −1.35 V vs. SCE were recorded in saturated N_2_ electrolyte at 5 mV s^−1^ varying the rotation rate from 1600, 900, 400, 225 to 100 rpm; (iii) saturation of the electrolyte with O_2_ and the procedures (i) and (ii) were performed again. The ring potential was set at a constant value of 1.2 V vs. SCE to oxidize the H_2_O_2_ formed on the disc electrode, allowing for its quantification. Before the LSV experiments, the Pt ring was activated by cyclic voltammetry in saturated N_2_ electrolyte at 500 mV s^−1^ (15 CV) from 0.4 V to −0.9 V vs. SCE. 

To calculate the number of electrons involved during ORR and the percentage of electrogenerated H_2_O_2_, Equations (3) and (4) were used, respectively.

### 3.3. Preparation and Electrochemical Characterization of EGO-ABA Based Electrodes for Supercapacitors

Nickel foams (NF) were used as support for the fabrication of EGO and EGO-ABA electrodes. The EGO-based materials and PTFE binder (90:10 wt.% ratio, 90 mg of graphene materials and 10 mg of PTFE) were mixed in a small volume (2 mL) of ethanol under sonication until a homogenized dispersion is obtained. Then, the Ni foam (1 cm^2^) was coated by simple repetitive dip-coating steps into EGO inks and dried at 60 °C under vacuum overnight. The active material loading (around 1.5–2.0 mg) was calculated from the weight difference of the nickel foam before and after coating determined by high precision weighing balance (accuracy 0.0001 mg). 

The electrochemical behavior of EGO-based NF electrodes was investigated by cyclic voltammetry in a three-electrode configuration, where carbon paper (CP) and a saturated calomel electrode (SCE) were employed as counter and reference electrodes, respectively. The electrochemical studies were carried out in 6 M KOH electrolyte and the scan rate varied between 5 and 100 mV s^−1^ [65]. The specific capacitance (Cs) of EGO/NF and EGO-COOHs/NF was evaluated from the cyclic voltammograms using the cathodic voltammetric charge (Q) integrated in the 0 to −1.15 V vs. SCE potential window by using Equation (5) [37]:

(5) $$Cs=\frac{Q}{m\Delta V}$$

where *Cs* is the specific capacitance (in F g^−1^), Q is the charge (in C), Δ*V* is the potential window (in V), and m is the mass of active material (in g).

## 4. Conclusions

In this work, we have synthesized the aminobenzoic acid functionalized electrochemically exfoliated graphene oxide (EGO-ABA) by one-step method. The amount of 4-aminobenzoic acid (4-ABA) in the 0.1 M sulfuric acid electrolyte was varied between 0 and 40 mM during the electrochemical exfoliation of graphite to obtain EGO materials with different extents of functionalization. It was found that the presence of 4-ABA decreases the rate of exfoliation and that several reactions occur simultaneously and compete with each other: (1) electrochemical oxidation of graphite vs. oxidation of 4-ABA; (2) the formation of EGO-ABA through grafting vs. the formation of azo dye as a side product. The concentration of 4-ABA should be limited to 10 mM to favor the grafting of 4-ABA on the EGO surfaces.

By adding different concentrations of 4-ABA, the EGO-ABA materials with different amounts and types of functional groups were obtained. Additionally, the EGO-ABA materials have more functional groups, higher density of defects, and a smaller size compared to EGO. 

The electrochemical performance of the EGO and EGO-ABA materials for oxygen reduction reaction (ORR) and for supercapacitors were explored in alkaline medium. The presence of the functional groups associated with smaller flake sizes increases the activity for ORR (lower onset potentials, shift of the polarization curves to more positive potentials and higher current) and the specific capacitance of the EGO. However, the non-modified EGO material has a higher selectivity for the 2-electron process compared to the EGO-ABA materials. This was attributed to a lower amount of carbonyl groups in the EGO compared to EGO-ABA. The EGO-ABA synthesized from 5 mM 4-ABA (EGO-ABA-5) has the highest specific capacitance than the others AGO-ABA materials and a remarkable cycling capability (80.6% retention of the initial capacitance after 5000 cycles at 50 mVs^−1^). It is concluded that the EGO-ABA materials synthesized from higher concentrations of 4-ABA (20 and 40 mM) have more unreacted 4-ABA and side products adsorbed on the EGO flakes’ surface hindering their electrochemical performance. Therefore, low concentration of amines should be used in this type of synthesis of EGO materials.

## Figures and Tables

**Table 1:** Experimental observations during the synthesis of the EGO and EGO-ABA materials.

Samples	[4-ABA] (mM)	I_ini_ (A)	t_exf_ (min)	Observation
EGO	0	1.67	60	Electrolyte colorless, graphite foil completely exfoliated
EGO-ABA-5	5	1.20	180	The electrolyte first turns purple around the graphite foil and then turns all brown; graphite foil completely exfoliated
EGO-ABA-10	10	1.14	180	The electrolyte first turns purple around the graphite foil and then turns all brown; graphite foil completely exfoliated
EGO-ABA-20	20	1.06	210	The electrolyte first turns purple around the graphite foil and then turns all brown; graphite foil almost completely exfoliated
EGO-ABA-40	40	0.90	210	The electrolyte first turns purple around the graphite foil and then all brown; graphite foil was not completely exfoliated

4-ABA: 4-aminobenzoic acid; I_ini_: initial current; t_exf_: exfoliation time.

## Data Availability

The data is available upon request.

## Supplementary Materials

The following supporting information can be downloaded at: https://www.mdpi.com/article/10.3390/molecules27217629/s1, Materials and Instrumentation; Figure S1. FT-IR spectra: (a) EGO and EGO-ABA materials, (b) 4-ABA; Figure S2. XPS spectra of C 1s (a) and N 1s (b) for EGO and EGO-ABA materials; Figure S3. SEM images for EGO and EGO-ABA-5 materials; Figure S4. (a) Cyclic voltammetry of the EGO and EGO-ABAs modified electrodes in N_2_-saturated 0.1M KOH solution after 20 cycles, scan rate 50 mV s^−1^. (b) Cyclic voltammetry of the GCE in N_2_-saturated 0.1M KOH solution with different concentration of 4-ABA after 20 cycles, scan rate 50 mV s^−1^; Figure S5. The faradic charge (Q_Faraday_) for the EGO and EGO-ABAs electrodes calculated from the CV recorded in N_2_-saturated 0.1M KOH solution; Figure S6. Cyclic voltammograms of Nickel foam (NF) and EGO-ABA-5/NF recorded at the scan rate of 50 mV s^−1^ from 0 to −1.15 V; Figure S7 Cyclic voltammograms recorded in 6 M KOH at different scan rates for each EGO-ABA/NF electrode; Table S1. Position of the 2D band in the Raman spectra for EGO and EGO-ABA materials.

## Sample Availability

Samples of the compounds are available from the authors.
