# Supplementary material for: One-Step Synthesis of Aminobenzoic Acid Functionalized Graphene Oxide by Electrochemical Exfoliation of Graphite for Oxygen Reduction to Hydrogen Peroxide and Supercapacitors
Source: Molecules. 2022 Nov 7;27(21):7629. doi: 10.3390/molecules27217629 (PMC9655138; doi:10.3390/molecules27217629)
Supplement: Supplementary file 1 [file molecules-27-07629-s001.zip › molecules-1942633-supplementary.pdf]

Supporting Information

One-step synthesis of amino benzoic acid functionalized graphene oxide by electrochemical exfoliation of graphite for oxygen reduction to hydrogen peroxide and supercapacitors

Yuting Lei [1†], Ludmila dos Santos Madalena [1,2†], Benjamin D. Ossonon [1], Fausto Eduardo Bimbi Junior [2], Jiyun Chen [1], Marcos R. V. Lanza [2] and Ana C. Tavares [1*]

[1] Institut National de la Recherche Scientifique – Énergie Matériaux Télécommunications (INRS-EMT), 1650 Boulevard Lionel-Boulet, Varennes, Québec, Canada, J3X 1P7

[2] Instituto de Química de São Carlos, Universidade de São Paulo, Av. Trab. São Carlense, 400 - Parque Arnold Schimidt, São Carlos, SP, 13566-590, Brazil

† These authors contributed equally to this work.

* Correspondence: ana.tavares@inrs.ca; Tel.: +1-514-228-6947

Contents

Materials and Instrumentation 3

Figure S1. 6

Table S1... 7

Figure S2. 7

Figure S3. 8

Figure S4. 8

Figure S5. 9

Figure S6. 9

Materials and Instrumentation

Materials. Graphite foil (0.5 mm thick, 99.8%) and 4-aminobenzoic acid ($C_7H_7NO_2$, 99%) were purchased from Alfa Aesar. Millipore water (18.2 MΩ cm) was used for rinsing the samples of EGO materials and for preparation of all aqueous solutions. Sulfuric acid (H_2SO_4, 95.0 - 98.0%) was purchased from Sigma-Aldrich. Potassium hydroxide (>99.99%), isopropyl alcohol (99.5%) and ethanol (91.5%) were purchased from Fisher Scientific and were used without further treatment. The 5 wt% Nafion® suspensions were purchased from Ion Power, Inc. Poly(tetrafluoroethylene) (PTFE) binder was purchased from Aldrich Chemical Company, Inc. High grade nickel foam (99.8% purity) was purchased from KUNHEWUHUA, China.

Instrumentation

The electrochemical exfoliation of graphite was conducted with a DC Power supply (HY3005F-3) from Dr. Meter.

Scanning Electron Microscopy (SEM) images were taken with a Tescan Vega 3 microscope operating at 20.0 keV incident energy.

Fourier Transform Infrared (FT-IR) spectra of the powder samples were recorded in the region 4000–600 cm^{-1} on a Nicolet FT-IR spectrophotometer.

The bulk structure of electrochemically exfoliated graphene oxide was characterized by X-ray diffraction (XRD) on Bruker D8 X-ray diffractometer with Cu-Kα radiation (λ = 1.54178 Å).

Raman spectra were recorded on a Raman microscope (Renishaw, inVia, at University of Québec at Montreal) with a laser source of 532 nm. The laser beam was focused on the sample on a spot size of 1 μm in diameter, and at least 5 spectra were recorded for each EGO film.

X-ray photoelectron spectroscopy (XPS) was performed with a VG Escalab 200i-XL equipped with a hemispherical analyzer (pass energy = 20 eV) and a multi-channel detector, applying a Twin Anode X-Ray Source at 15 kV and 20 mA. The base pressure inside the spectrometer during analysis was less than 7 × 10-10 torr. The binding energy of the C 1s peak at 284.5 eV was used as internal standard. The core level spectra were peak-fitted using Lorentzian and Gaussian curves after the Shirley type background subtraction, and Casa XPS software. Peak areas were normalized by appropriate atomic sensitivity factors.

A Princeton applied research 273A and an Autolab PGSTAT128N potentiostats were used to conduct the electrochemical tests. A glassy carbon electrode (GCE, 5 mm of diameter, from Pine Instrument Co), a glassy carbon rotating ring disk RRDE (AFE7R9GCPT, Pine Instrument Co) and controller (AFMSRCE, Pine Instrument Co.) were used for the cyclic voltammetry and oxygen reduction studies in 0.1M KOH. The parameters of the RRDE are the following: **e**lectrode area (disk): 0.2472 cm^{-2}, ring-disk gap = 320 μm; collection efficiency = 37%; disk OD = 5.61 mm; Ring OD = 7.92 mm; Ring ID = 6.25 mm.

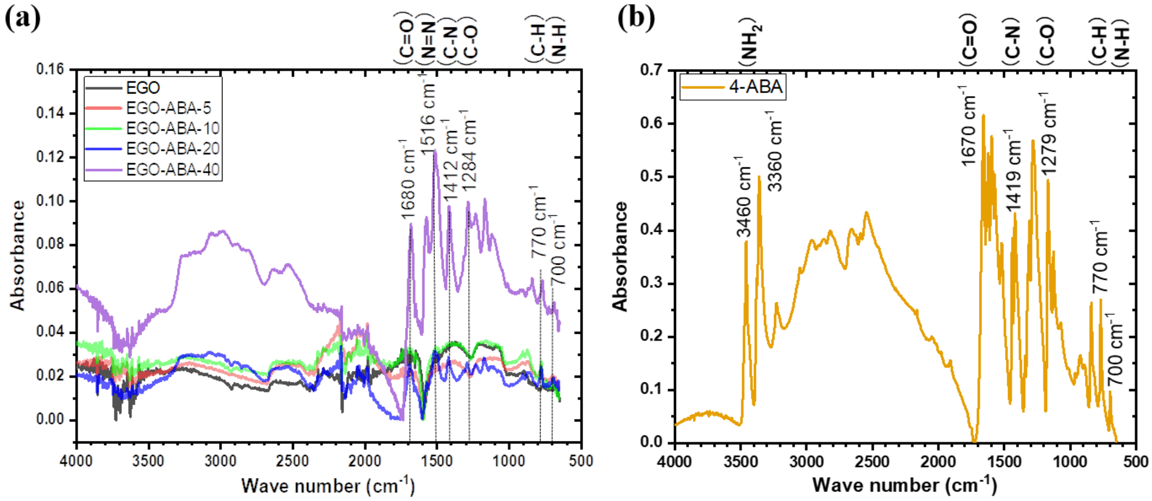

Figure S1. FT-IR spectra: (a) EGO and EGO-ABA materials, (b) 4-ABA.

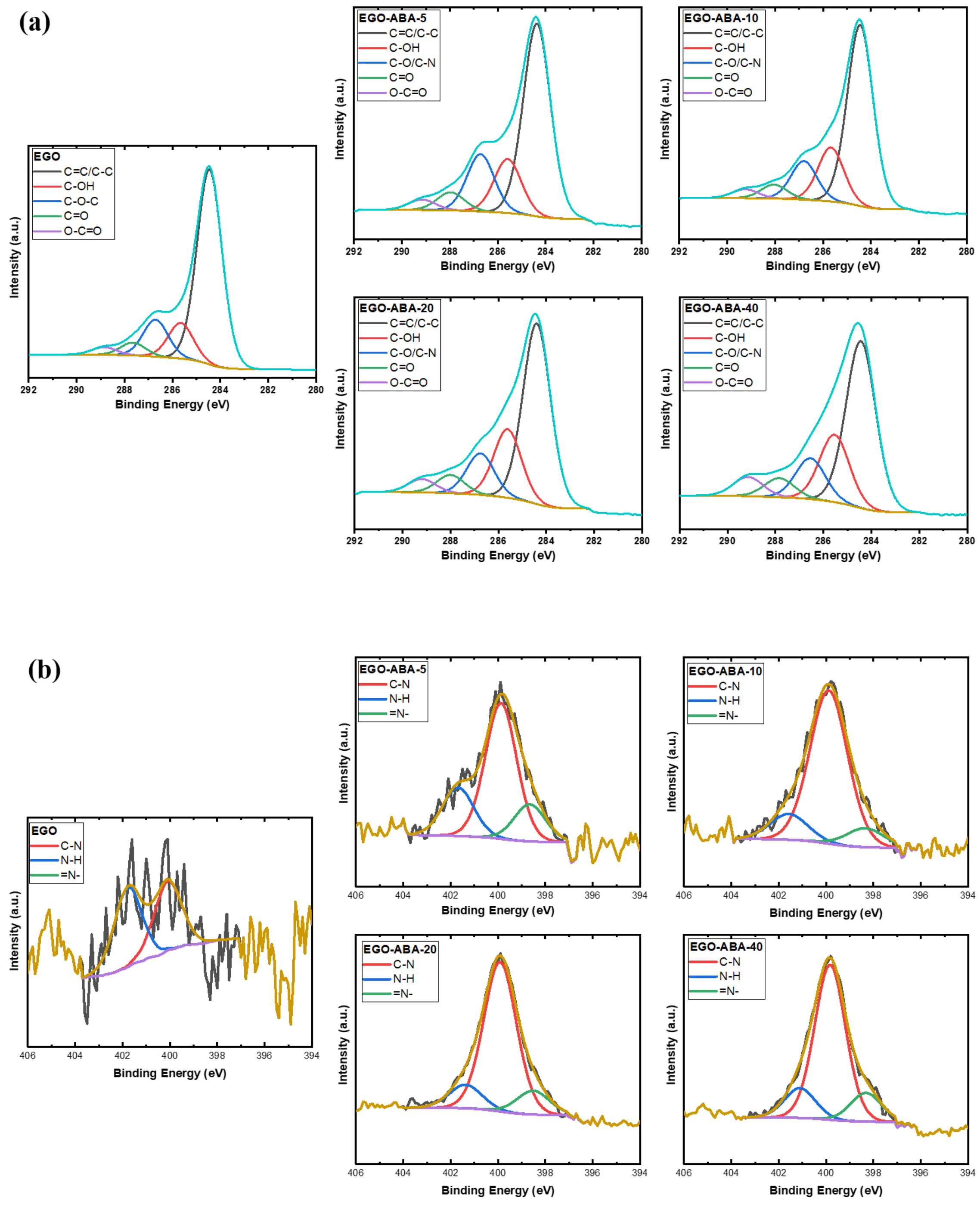

Figure S2. XPS spectra of C 1s (a) and N 1s (b) for EGO and EGO-ABA materials.

Table S1. Position of the 2D band in the Raman spectra for EGO and EGO-ABA materials

Samples	2D (cm^{-1})	Standard deviation (cm^{-1})
EGO	2702	9
EGO-ABA-5	2705	5
EGO-ABA-10	2697	10
EGO-ABA-20	2697	5
EGO-ABA-40	2705	4

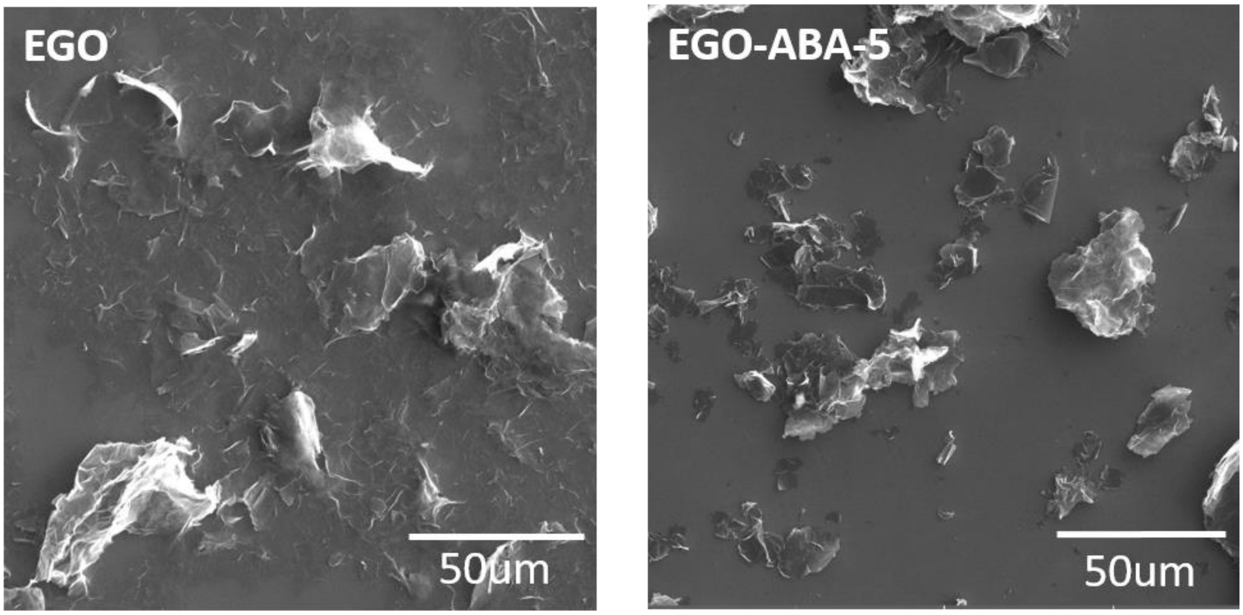

Figure S3. SEM images for EGO and EGO-ABA-5 materials.

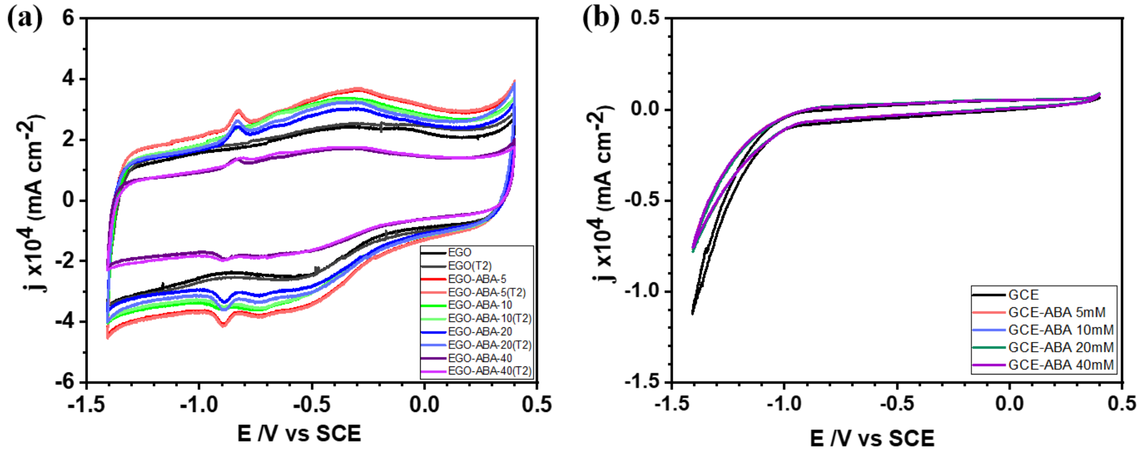

Figure S4. (a) Cyclic voltammetry of the EGO and EGO-ABAs modified electrodes in N_2-saturated 0.1M KOH solution after 20 cycles, scan rate 50 mV s^{-1}. (b) Cyclic voltammetry of the GCE in N_2-saturated 0.1M KOH solution with different concentration of 4-ABA after 20 cycles, scan rate 50 mV s^{-1}.

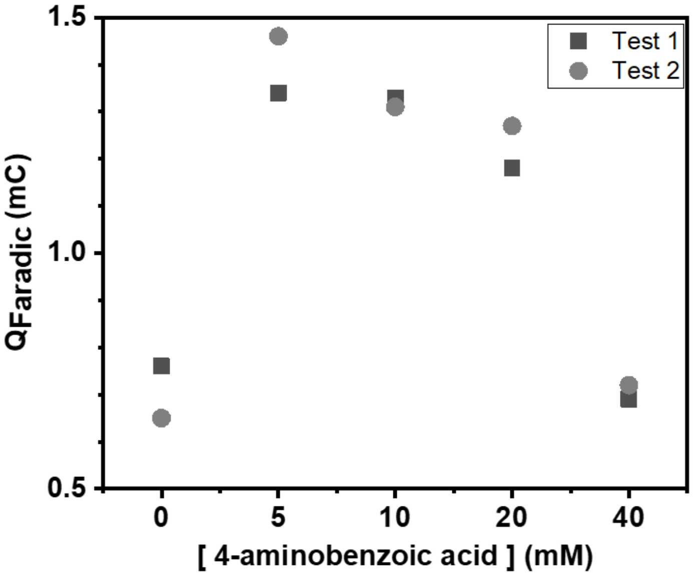

Figure S5. The faradic charge ($Q_{Faradic}$) for the EGO and EGO-ABA electrodes calculated from the CV recorded in N_2-saturated 0.1M KOH solution.

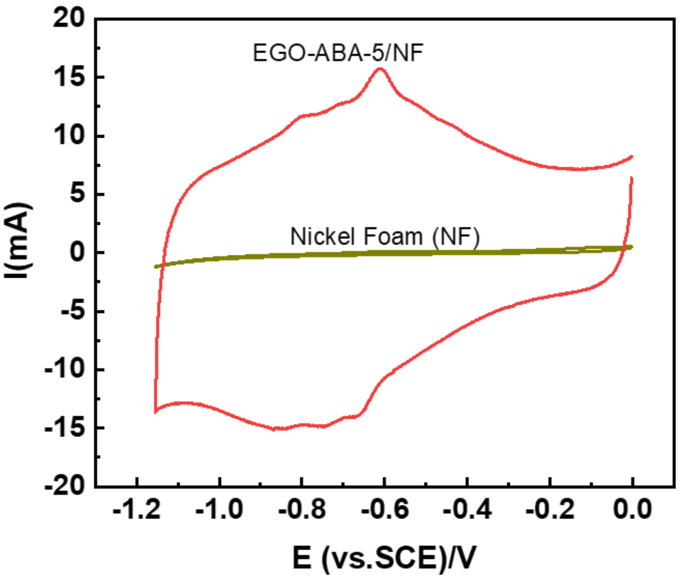

Figure S6. Cyclic voltammograms of Nickel foam (NF) and EGO-ABA-5/NF recorded at the scan rate of 50 mV s^{-1} from 0 to -1.15 V.

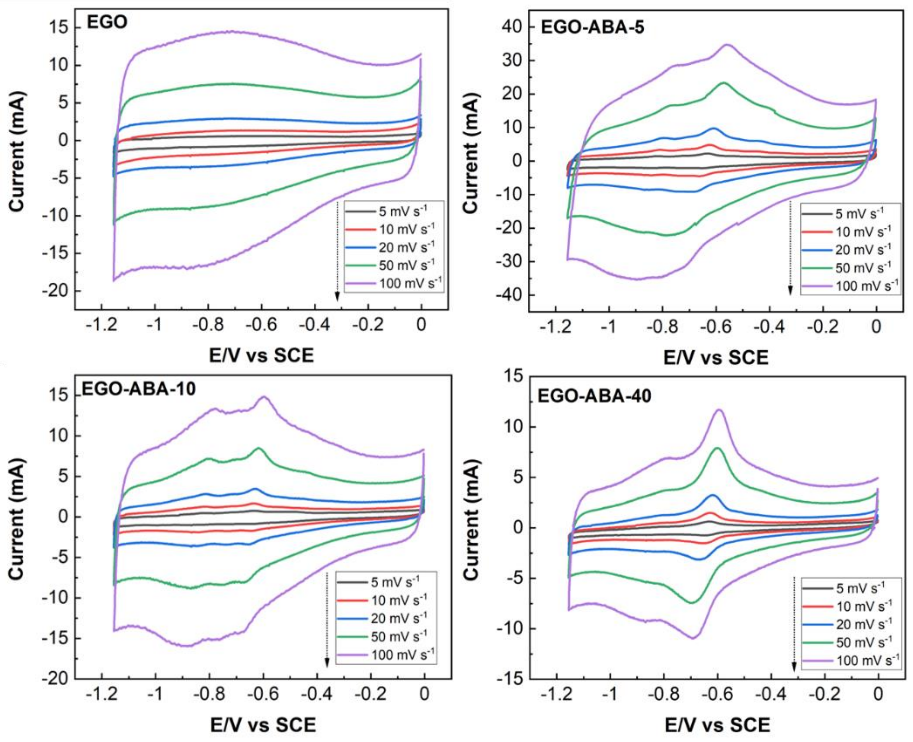

Figure S7. Cyclic voltammograms recorded in 6 M KOH at different scan rates for each EGO-ABA/NF electrode.
